# Supplementary material for: Two-pore channel blockade by phosphoinositide kinase inhibitors YM201636 and PI-103 determined by a histidine residue near pore-entrance
Source: Commun Biol. 2022 Jul 23;5:738. doi: 10.1038/s42003-022-03701-5 (PMC9308409; doi:10.1038/s42003-022-03701-5)
Supplement: Supplementary file 2 — Description of Additional Supplementary Files [file 42003_2022_3701_MOESM2_ESM.pdf]

Description of Additional Supplementary Files

File Name: Supplementary Data

Description: Source data
